# Welcome to *Investigative Genetics*

**DOI:** 10.1186/2041-2223-1-1

**Published:** 2010-09-01

**Authors:** Manfred Kayser, Bruce Budowle, Antti Sajantila

**Affiliations:** 1 Department of Forensic Molecular Biology, Erasmus University Medical Centre Rotterdam, Rotterdam, Netherlands; 2 Department of Forensic and Investigative Genetics, University of North Texas Health Science Center, Texas, USA; 3 Hjelt Institute, Department of Forensic Medicine, University of Helsinki, Helsinki, Finland

## 

The initial questions often asked when a new journal appears are, "What is different about this journal compared with existing ones?", and "Why should I contribute to this journal?" These questions are becoming even more relevant with the appearance of many new journals in various areas of science and a substantial contribution from online-only publishers, because the forum in which one publishes one's work impacts its value, distribution, and communication. We ourselves, now bringing you the journal *Investigative Genetics*, asked the same questions, and initially had reservations about creating the journal. Could this new journal make a meaningful contribution to the scientific community? Currently, there are many peer-reviewed scientific journals (established ones as well as newly-initiated ones) that cover the field of molecular genetics from various basic and applied aspects, and more recently, with advances in DNA technology, genomics journals have also been founded. Hence, we saw little value in simply adding another journal to the genre. Instead, with our new initiative, we aim to provide a platform for publishing results from molecular genetic and genomic research in areas with societal relevance. There is a whole spectrum of societal implications that spring from molecular genetic and genomic research, ranging from forensic issues and legal medicine, to evolutionary biology, anthropological and historical studies, epidemiology and medical applications, and biosafety. Although existing genetics and genomics journals do publish papers on such topics--together with many other more basic aspects--to the best of our knowledge, a journal dedicated to molecular genetics and genomics with societal relevance does not exist, at least not with the wide range of interrelated topics we anticipate covering with our initiative. While the subject matter that molecular genetic and genomic studies address may vary, there are substantial commonalities in the applied study designs, technologies, analytical approaches, as well as interpretation of results in these studies. We believe that scientists from various fields allied by their application of molecular genetic and genomic principles and techniques to scientific questions of societal relevance will benefit and be able to leverage knowledge and other resources from a journal providing an integrated platform.

*Investigative Genetics *provides an open access, online-only highly visible venue for novel cutting-edge research and technological developments, which through the application of molecular genetics and genomics, answer questions about past and present life, enhance life quality, and provide a safer living environment. The journal aims to reflect the breadth of molecular genetic and genomic research, its development and validation, and its application to a greater understanding, improvement in quality of life, and enhanced safety in our society. It bridges the gap between different areas of science that similarly exploit molecular genetics and genomics, allowing findings and developments to be disseminated efficiently and effectively across disciplines. Articles published in this journal will be hypothesis-driven and will apply, if appropriate, statistical principles (purely descriptive studies will be of limited interest to the journal). The main areas of interest include forensic genetics, biosafety and biosecurity, synthetic biology, legal medicine, genetic epidemiology, identity and lineage testing, personalized genetics and genomics, population genetics and genomics, evolutionary genetics and genomics, mechanisms of inheritance, ancient bio-materials, anthropological genetics and genomics, and archaeogenetics and archaeogenomics. In addition, the journal will publish articles addressing the important issues of statistics, validity, reliability, and ethics related to the use and understanding of genetics and genomics. We are also pleased to announce a special contribution in the form of a regular commentary from Professor Mark Jobling [1]. His insight and depth of knowledge will make for interesting and exciting thought-provoking discussion in many of the areas covered by *Investigative Genetics*.

*Investigative Genetics *is considering for fast and thorough peer-review two types of original research papers that will form its major content. Research Reports: full-length articles reporting data from original research; and Short Reports: brief articles on original research findings. In addition, the journal is considering Methodology Articles: presentations of experimental methods, tests or procedures, providing that they are novel or may offer a better version of an existing method, which will be fully peer-reviewed. Furthermore, the journal publishes three types of articles invited by the Editors (and the Editors-in-Chief will consider suggestions for invitations). Reviews: fully peer-reviewed comprehensive, authoritative discussions of any subject within the scope of the journal. Opinions: pieces on specific scientific topics, including controversial ones, not fully summarizing a field but rather providing an author's own scientific viewpoint. Commentaries: updates of recent papers within the scope of the journal that have been published elsewhere. The latter two types of articles are not peer-reviewed, as they represent, at least in part, personal views on scientific research. Finally, we consider Letters to the Editors: brief comments, including critical ones, about articles recently published by the journal (other topics will not be considered in this format), which will be peer- reviewed, and the author(s) of the initial article will be given the possibility of responding.

We are delighted that the Editorial Board [2] of *Investigative Genetics *comprises distinguished experts in all the different areas covered by the journal's scope, and we would personally like to thank all colleagues for kindly agreeing to serve as Editorial Board members. We are committed to developing a high-quality journal that provides important findings and addresses topical issues in a timely fashion, and we look forward to contributions of novel research, and thoughtful opinions, reviews or commentaries.

*Investigative Genetics *can only be successful if the scientific community shares our vision. We look forward to receiving many interesting and high-quality manuscripts for publication, a successful start, and a prosperous life for the journal.

## References

[B1] Jobling M Tales the double helix tells Invest Genetic 2010 1 2 10.1186/2041-2223-1-2 PMC2988478 21092336

[B2] Investigative Genetics Editorial Board http://www.investigativegenetics.com/about/edboard

